# Supplementary material for: The relationship between physical education teachers’ competence support and middle school students’ participation in sports: A chain mediation model of perceived competence and exercise persistence
Source: PLoS One. 2025 Jan 7;20(1):e0314338. doi: 10.1371/journal.pone.0314338 (PMC11706385; doi:10.1371/journal.pone.0314338)
Supplement: Attachment — Submitted filename: Response to Reviewers.docx [file pone.0314338.s002.docx]

Response to Reviewers

Thank you to the two reviewers for their valuable suggestions on improving the manuscript. We have responded to journal requirements and each comment and made all of the suggested changes. The paper was also proofread to avoid grammatical errors.

Journal Requirements:

1.Please ensure that your manuscript meets PLOS ONE's style requirements, including those for file naming.

Response 1: We revised the formatting requirements and file naming requirements of the manuscript to comply with the journal's requirements.

2. We suggest you thoroughly copyedit your manuscript for language usage, spelling, and grammar.

Response 2: We re-edited the manuscript for language use, spelling, and grammar to meet the journal's requirements.

3. Data Availability

Response 3: We uploaded the data to the journal's designated database, DRYAD, DOI: https://doi.org/10.5061/dryad.brv15dvkd. and uploaded the data as a support information file.

Reciew#1
Comment 1. Pay attention to the accuracy of citations.
For example, the author said Teacher support, an important external environmental factor widely advocated in self-determination theory, is generally considered from the perspective of students [6].

Self-determination theory has never emphasized teacher support; it has only highlighted the external environment. Teacher support is a significant part of the external environment. Therefore, when discussing this concept, it should be stated that teacher support, as an integral part of important social support or as a crucial environmental variable, plays a significant role in the development of students' capabilities. The author's current phrasing should be avoided, as there are many similar sentences throughout the text.

Response 1: We really appreciate your comments on this matter. We reorganized the relationship between teacher support and self-determination theory and revised this section in the text. Please see L102-L107.

Comment 2. The entire text must be polished, especially in terms of expression, where many parts can lead to ambiguity, as exemplified earlier. Additionally, there are traces of awkward translations from Chinese to English in several places.

Response 2: We have revised the article to address some of the problems that existed in terms of expression, as well as traces of insufficient fluency in Chinese and English translations, and we have embellished the entire article.

Comment 3. Sentences should generally not be too long. For instance, the first sentence of the abstract spans 4-5 lines, which could be broken down for clarity.

Response 3: We broke up sentences that were too long in the article to improve overall clarity. Please see L13-L19.

Comment 4.  Please carefully refer to high-level publications, such as quantitative research papers published in this journal, to revise the terminology throughout the paper. For example, the third section should be titled "Methodology," and research tools should be referred to as "instruments" or "measures."
Response 4: We referenced high-level publications and revised the terminology in the article. Please see L225 and L242.

Comment 5. Consistency is required when reporting data. For example, the chi-square and degrees of freedom ratio can be reported together, but the author uses separate expressions when reporting the KMO value corresponding to the chi-square degrees of freedom ratio for the teacher support dimension.

Response 5: We modified the reporting of chi-square values and degrees of freedom ratios to ensure consistent data reporting. Please see L261-L265, L270-L274, L283-L287.

Comment 6. The author should clearly explain the meaning of several core variables in the literature review, such as what constitutes teacher support and student ability. Additionally, "teacher support" should be referred to as" students' perceived teacher support," as it is measured and perceived from the student's perspective.
Response 6: We added explanations of several core variables to the literature review and revised the section where “teacher support” should be called “students' perceived teacher support.” Please see L49, L310, L312, L314, L315, L334, L349, L357.

Comment 7. There should be a corresponding theoretical discussion on why the current topic is researched from a particular angle.

Response 7: We added a theoretical foundations section as a way to ensure a specific perspective on the research topic. Please see L77-L98.

Comment 8. The expression of the research gap is not particularly accurate, and there is a lack of effective discussion on the purpose of the research.

Response 8: We added a current research gap section to ensure effective discussion of the research hypothesis and research objectives. Please see L127-L134, L160-L167, L193-L197, L202-L211.

Comment 9.  Interdisciplinary research has certain guiding significance for this study; please refer to the following literature.
Response 9: We have carefully referenced several of the papers you submitted and similar interdisciplinary research has been very enlightening for this study. We have added a number of interdisciplinary themes to our study and have made modifications to our research. For example:

[1] Reeve, J., & Jang, H. (2006). What teachers say and do to support students' autonomy during a learning activity. Journal of educational psychology, 98(1), 209.

[2] Ryan, R. M., & Deci, E. L. (2000). Self-determination theory and the facilitation of intrinsic motivation, social development, and well-being. American psychologist, 55(1), 68.
[3] Skinner, E. A., & Belmont, M. J. (1993). Motivation in the classroom: Reciprocal effects of teacher behavior and student engagement across the school year. Journal of educational psychology, 85(4), 571.

Review#2

**INTRODUCTION**

Comment a. Broaden the literature review to include more diverse studies and recent developments in the role of teacher support in sports participation.

Response a: We have added recent advances and diverse research on teacher support in sport participation. Please see L47-L53.For example:

[1]Ghorbani S, Nouhpisheh S, Shakki M. Gender differences in the relationship between perceived competence and physical activity in middle school students: Mediating role of enjoyment. International journal of school health. 2020;7(2):14-20.

[2] Eberline A, Judge LW, Walsh A, Hensley LD. Relationship of enjoyment, perceived competence, and cardiorespiratory fitness to physical activity levels of elementary school children. Physical Educator. 2018;75(3):394-413.

Comment b. Strengthen the link between the conceptual framework and the stated hypotheses to clarify the study's novelty and theoretical contribution.

Response b: We strengthened the link between the conceptual framework and the research hypothesis. Please see L63-L65.

**LITERATURE REVIEW AND HYPOTHESES**

Comment a. Expand the discussion of the chain mediation effect, providing more theoretical grounding for how perceived capability and exercise persistence jointly influence sports participation.

Response a: We extend the discussion of chain mediation effects and add a Theoretical Foundation section. Please see L77-L98,L202-L211.

Comment b. Ensure that all hypotheses are clearly tied to specific gaps in the literature to highlight their significance

Response b: We have added a description of the research gaps in the literature review section, highlighting the links between the research hypotheses and specific research gaps. Please see L127-L134, L160-L167, L182-L185,L193-L197.

**METHODS**

Comment a. Acknowledge the geographic and demographic limitations of the sample and their implications for generalizability.

Response a. We added geographic and demographic limitations to the sample and placed this section in the Research Limitations section. Please see L686-L713.

Comment b. Discuss the limitations of cross-sectional design and suggest longitudinal or experimental approaches for future research.

Response b: Similarly, we add a discussion of the limitations of the article's cross-sectional design and suggest a longitudinal or experimental approach for future studies. Please see L686-L713.

**RESULTS**

Comment a. Consolidate the presentation of results to eliminate repetitive descriptions.

Response a: We simplified the reporting of descriptive findings. Please see L358-L362, L364-L368.

Comment b. Use visuals to succinctly illustrate key findings, such as the mediation pathways and model fit indices.

Response b: We added visual tools for mediating paths and model fit indices. Please see L380.

**DISCUSSION**

Comment a. Expand on the practical implications of the findings, particularly for teacher training and curriculum design.
Response a: We increased the practical implications of the findings in order to facilitate an increase in the impact of this study on physical education teaching and learning. Please see L448-L475, L515-L537, 570-L584.

Comment b. Focus on critical insights and avoid restating results already presented.

Response b: We have removed some duplicate results and added unique concerns. Please see L448-L475, L515-L537, 570-L584.

**CONCLUSION**

Comment a. Include specific recommendations for physical education practices, such as strategies for enhancing teacher support and fostering student engagement in sports.

Response a: We have added a number of descriptions of the findings to extend the practical recommendations of this study for teaching and learning in physical education. Please see L645-L659.

Comment b. Highlight potential areas for future research, such as examining other mediators or exploring interventions in different cultural contexts.

Response b: We add descriptions of potential areas similar to this study. Please see L645-L659.

**LIMITATIONS**

Comment a. Expand on the limitations related to the study design and sample characteristics.

Response a: We extend the description of limitations related to study design and sample characteristics. Please see L686-L713.

Comment b. Suggest directions for future research, such as incorporating longitudinal designs or exploring additional influencing factors.

Response b: We have added a description of future research directions. Please see L786-L713.
